# Faecal bifidobacteria in Indian neonates & the effect of asymptomatic rotavirus infection during the first month of life

**Published:** 2010-12

**Authors:** Ramadass Balamurugan, Fabien Magne, Divya Balakrishnan, Antonia Suau, Sasirekha Ramani, Gagandeep Kang, Balakrishnan S. Ramakrishna

**Affiliations:** Department of Gastrointestinal Sciences, Christian Medical College, Vellore, India; * Laboratoire de Biologie, Paris, France

**Keywords:** Bifidobacterium, colonization, gut, neonates, rotavirus

## Abstract

**Background & Objectives:**

Bifidobacteria colonize the gut after the first week of life and remain an important component of the gut microbiota in infancy. This study was carried out to characterize the diversity and number of bifidobacteria colonizing the gut in Indian neonates and to investigate whether asymptomatic infection with rotavirus in the first month of life affected gut colonization by bidifobacteria.

**Methods:**

DNA was isolated from faeces of 14 term-born neonates who were under surveillance for rotavirus infection. Bacterial and bifidobacterial diversity was evaluated by temporal temperature gradient electrophoresis (TTGE) of 16S rDNA amplified using total bacteria and bifidobacteria-specific primers. Real time PCR, targeting 16S rDNA, was used to quantitate faecal bifidobacteria and enterobacteria.

**Results:**

TTGE of conserved bacterial 16S rDNA showed 3 dominant bands of which *Escherichia coli* (family *Enterobacteriaceae*) and *Bifidobacterium* (family *Bifidobacteriaceae*) were constant. TTGE of *Bifidobacterium* genus-specific DNA showed a single band in all neonates identified by sequencing as *Bifidobacterium longum* subsp. *infantis*. Faecal bifidobacterial counts (log_10_ cfu/g faeces) ranged from 6.1 to 9.3 and enterobacterial counts from 6.3 to 9.5. Neonates without and with rotavirus infection in the first week of life did not show significant differences in the median count of bifidobacteria (log_10_ count 7.48 vs. 7.41) or enterobacteria (log_10_ count 8.79 vs. 7.92).

**Interpretation & Conclusions:**

*B. longum subsp. infantis* was the sole bifidobacterial species colonizing the gut of Indian neonates. Asymptomatic rotavirus infection in the first month of life was not associated with alteration in faecal bifidobacteria or enterobacteria.

The human gut is sterile at birth, and microbial colonization commences during or immediately after delivery^1^. Facultative anaerobes appear first, consisting of Enterobacteria, *Streptococcus* and *Staphylococcus. Bifidobacterium* and *Lactobacillus* species appear after the first weeks of life and the former constitute the predominant bacterial species in the infant gut^2^–^4^. Studies in other countries indicate that a variety of bifidobacteria may be found in the faecal flora of infants; greater than 40 per cent were uncharacterized in one study, while *Bifidobacterium infantis* and *Bifidobacterium breve* were the next most common species found^5^. Bifidobacteria are believed to be beneficial to health. Interventions to increase their population by administering them as probiotics, or administering prebiotics to stimulate their growth and colonization, are often suggested. It is also suggested that bifidobacteria protect against or attenuate rotaviral illness in infants. Studies in experimental animals indicate that administration of bifidobacteria resulted in the development of increased titres of IgA antibody in both faeces and serum of infected animals, indicating that the bacteria potentiated immune responses^6^. Administration of bifidobacteria to infants and young children reduced subclinical rotavirus shedding as indicated by development of salivary antibodies^7^. Administration of bifidobacteria to infants and young children either in the community^8^ or those with acute diarrhoea^9^ reduced faecal shedding of rotavirus in the treated groups.

Bifidobacterial colonization of the gut in early infancy may be affected by a variety of factors including the mode of delivery, type of feeding, and antibiotic use^1^,^4^,^5^. Asymptomatic infection with rotaviruses is well described in neonates especially in the setting of neonatal nurseries^10^–^14^. In an investigation undertaken in the Department of Gastrointestinal Sciences, Christian Medical College, Vellore, samples of faeces were collected longitudinally from neonates for over a month and the main result was reported elsewhere^13^. There is presently no information on the species diversity or numbers of bifidobacteria in the intestinal flora of Indian infants. It is also not known whether infection with an enteric virus such as rotavirus, during the critical first month of life, can affect colonization of the gut with bifidobacteria. As the samples from the earlier study could potentially answer both questions, we designed a study utilizing the already collected faecal samples to characterize bifidobacterial diversity and number in Indian neonates, and to investigate the effect of transient rotavirus infection in the first month of life on the faecal bifidobacterial population in Indian neonates. Total bacterial diversity and quantitation of Enterobacteriaceae were also undertaken as control for the effects on bifidobacteria.

## Material & Methods

The samples for this study were drawn from a study of molecular surveillance for rotavirus infections in the neonatal nursery of the Christian Medical College, a tertiary care hospital in Vellore, southern India, the primary results of which have been already reported^13^. In that study, faecal samples from neonates had been screened for rotavirus using an enzyme immunoassay (EIA) for detection of VP6 antigen (Rota IDEIA, Dako Ltd., UK). A subset of neonates resident in Vellore town had been enrolled in a longitudinal study to determine the pattern of rotavirus shedding, and were visited every day by a trained field worker to collect as many sequential samples as possible. Every child was followed for a minimum of 30 days. The study was approved by the Institutional Ethics Committee of the Christian Medical College, Vellore, and informed consent was taken from the parents for the primary study as well as subsequent analyses on collected faecal samples. The present investigation utilized fecal samples from 14 neonates, seven of whom did not have rotavirus detected in stool during the period of study and the remaining seven rotavirus had detected in the stool in the first week of life. For each participant in the latter (rotavirus-positive) group two faecal samples were examined, the first being one which had tested positive for rotavirus and the second collected 8-14 days later which was negative for rotavirus by EIA.

Stool was not cultured for bacteria. Bacteria were evaluated using molecular methods targeting conserved or variable regions of the 16S ribosomal DNA. Faecal DNA was extracted from 250 mg of the faeces using the QiaAMP Stool DNA minikit (Qiagen, Germany). DNA was checked for integrity and quantity and stored at -20°C until further analysis.

*Total bacterial temporal temperature gradient electrophoresis (TTGE)*: The primers S-D-Bact-339-a-S-20 and S-D-Bact-788-a-A-19 (Table) were used to amplify the variable regions 3 and 4 of the bacterial 16S rRNA genes. GC-rich sequence (5’-CCC CCC CCC CCC CGC CCC CCG CCC CCC GCC CCC GCC GCC C-3’) was added to the 5’end of the primer S-D-Bact-788-a-A-19 as previously described^15^. The Dcode universal mutation detection system (Bio-Rad, Hercules, CA) was used for sequence-specific separation of amplicons. Known bacterial strains were used to standardize band migration and gel curvature among different gels. This ladder consisted of the following organisms listed in their migration order: *Bacteroides sp., Enterococcus faecium, Staphylococcus epidermidis, Escherichia coli* and *Bifidobacterium longum*. TTGE gel patterns were analyzed using Diversity Database 2.1 Discovery Series (Bio-Rad, USA)^18^. Bands were detected automatically and defined through their relative intensity and relative front (Rf).

**Table:** Primers used in this study

Target	Primer	Reference	Sequence from 5’ to 3’ end	Tm
Bacteria	S-D-Bact-339-a-S-20	(Magne *et al*, 2006)^15^	CTC CTA CGG GAG GCA GCA GT	55°C
	S-D-Bact-788-a-A-19		GGA CTA CCA GGG TAT CTA A	
Bifidobacteria	S-G-Bif-164-a-S-18	(Satokari *et al*, 2001)^16^	GGG TGG TAA TGC CGG ATG	62°C
	S-G-Bif-662-a-A-18		CCA CCG TTA CAC CGG GAA	
Enterobacteria	Ent-1113	(Vasquez *et al*, 2010)^17^	UGG CAA CAA AGG AUA AGG	58°C
	Ent-1418		CUU UUG CAA CCA ACU	

*Bifidobacterial* TTGE: The forward primer Bif164f and reverse primer Bif662r linked to a GC clamp [CGC CCG CCG CGC GCG GCG GGC GGG GCG GGG GCA CGG GGG GCC ACC GTT ACA CCG GGA A]^16^ were used to amplify 16S rDNA of the *Bifidobacterium* genus from the samples. A PCR amplification of 35 cycles was done under the conditions previously described, with a lower hybridization temperature of 52°C, because of the GC clamp. The PCR products were separated on TTGE. Electrophoresis was performed in a 9 per cent polyacrylamide gel (160×160×1 mm) (37.5:1 acrylamide-bisacrylamide) and 8.5M urea (Interchim, Montluc, France). Premigration was realized at 20V and 66°C over a 15-min period. Gels were run overnight at 80V with the temperature increasing at 0.2°C from 66 to 70°C. The DNA fragments were visualized using SYBR Green I staining (Interchim, France) and the gel was scanned using Gel Doc 2000 (Bio-Rad, France). A standard reference was included every six lanes in each gel in order to normalize the gel profiles by comparison with a standard pattern. The normalization enabled comparison between TTGE profiles from different gels if these had been run under comparable conditions. Reproducibility of the technique was assured by running the same faecal samples several times on different days.

TTGE bands were excised from the gel and stored overnight at 4°C in 50 ml of sterile water. DNA was first amplified with primers Bif164f and Bif662rGC and the products checked on a TTGE gel. Next, amplifications were carried out using the primers Bif164f and Bif662r, and the products were sequenced, without cloning, using primer Bif662r (Sequentia, Clermont-Ferrand, France). These sequences were manually corrected using Chromas ver. 1.45 (Technelysium Pty. Ltd, Australia). A search of the GenBank nucleotide database was conducted using the BLAST algorithm to determine the closest relative of the partial 16S rRNA gene sequences.

*Real time PCR for quantitation of Enterobacteriaceae and Bifidobacteriaceae:* Real time PCR was used to quantify enterobacteria and bifidobacteria in stool samples. Primers were designed to amplify genus-specific segments of the 16S rDNA (Table). These primers have been extensively validated using *in silico* PCRs and by PCR against a variety of standard bacteria available in the laboratory and their use has been described earlier^15^,^18^,^19^. Real-time PCR was performed using the Mastercycler Ep Realplex (Eppendorf, France). The amplification reactions were carried out in a total volume of 25 µl containing 11.25 µl SYBR Green qPCR RealMaster Mix 2.5 × (Eppendorf), 0.4 µl of each probe (0.3 µM), 10.95 µl of sterile water and 2 µl of DNA samples. The reaction conditions for amplification of DNA were 96°C for 2 min, 40 cycles of 96°C for 15 sec, specific hybridization temperature(Table) for 1 min and 68°C for 4 min. To determine the specificity of amplification, analysis of product melting curve was performed after the last cycle of amplification. The dissociation curve obtained at the end of each PCR was checked and always had a similar melting point to the standard samples, without any additional peak, indicating the absence of non-desired PCR products. The melting curve was obtained by slow heating at temperatures from 60 to 96°C at a rate of 0.2°C/sec with continuous fluorescence collection. A negative control and a positive control were included on each plate. Each assay was performed in duplicate in the same run. The cycle threshold (Ct) was calculated as the cycle number at which the reaction became exponential.

Standard curves were constructed using plasmid containing 16S rDNA fragments amplified with corresponding primers. The plasmid concentration was measured using Qubit^TM^ fluorimeter (Invitrogen, USA) according to the manufacturer’s instructions. The number of copies was calculated as described earlier^19^. Serial dilutions were performed and 10^2^, 10^3^, 10^4^, 10^5^, 10^6^, 10^7^and 10^8^ copies of the gene per reaction were used for calibration. The standard curve was constructed comparing the cycle threshold of each sample with the number of copies. The quantities of target copies contained in an unknown sample were determined from the standard curve.

*Statistical analysis*: Bacterial counts were expressed as log_10_ counts/g faeces. Values are shown as median and interquartile range (IQR) in the text. The primary comparison was between neonates without (Control group) and with (Rota+ group) rotavirus infection. However, the second (Rota) group had two faecal samples examined, the first during active rotavirus infection (Rota+) and the second after clearance of infection (Rota-). Significance of differences between control and Rota+ groups was assessed using Mann Whitney U test for unpaired comparisons, and differences between Rota+ and Rota- samples were compared using the Wilcoxon matched pairs test for paired comparisons.

## Results

The neonates (7 male) had been delivered at term by normal vaginal delivery (11) or by Cesarean section (3) and did not have any evidence of congenital or acquired disease at the time of sampling. All the neonates were breast-fed. Half of them had evidence of rotavirus infection during the first month of life, while serial faecal testing in the other seven did not at any time reveal evidence of rotavirus infection. Neonates with rotavirus infection included in the present study never became symptomatic with diarrhoea. In the group of neonates with rotavirus infection, the median age at the time of detection of infection was 11 (range 6-30) days, and they excreted rotavirus in stool for a median of 5.5 (range 2-25) days. In this group of neonates, the first faecal sample was taken during the period (median 11 days, range 6-30 days) when the stool was positive for rotavirus (Rota+). A second faecal sample (Rota-) was taken when they had cleared the rotavirus infection, at a median of 17 (range 12-34) days of age. Faecal samples from neonates who did not have evidence of rotavirus infection at any time during the first month of life (Control) were obtained at median 12 (range 10-27) days after birth.

**Fig. 1:**
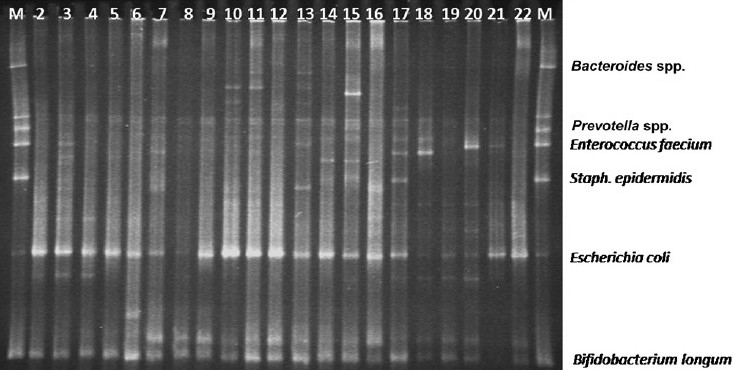
TTGE gel showing bands obtained by electrophoresis of PCR products from neonate faeces using primers targeted at conserved bacterial 16S rDNA sequences representative of domain bacteria. Lanes at right and left extreme are with marker DNA. Bands 63 (*Escherichia*) and 97 (*Bifidobacterium*) are marked. Lanes 1 & 23 are marker DNA; lanes 1, 4, 7, 10, 13, 15, 18, 21, 22 are samples from rotavirus - negative stool, while other lanes are from rotavirus-positive stool.

The gels resulting from PCR amplification of conserved bacteria domain sequences followed by TTGE (evaluation of total bacterial diversity) were examined for bands at 100 possible positions. Two bands, corresponding to *E. coli* (band 63) and *Bifidobacterium* genus (band 97), were almost constant being found in 14 and 12 neonates respectively. Other bands were less dominant (band 90 in 9 neonates, band 27 in 5 neonates, band 44 in 2 neonates, and bands 32, 33, 35, 37, 46, 73 in one neonate each) (Fig. 1). The median number of bacterial bands in individual neonates in the control group was 3 (IQR 3-4) which was not statistically significantly different from the median of 3 (IQR 2-4) in the Rota+ group. Among the Rota group, the number of bands after clearance of rotavirus infection (Rota-, median 4, IQR 3-8) was not different from the number during rotavirus infection (Rota+).

**Fig. 2:**
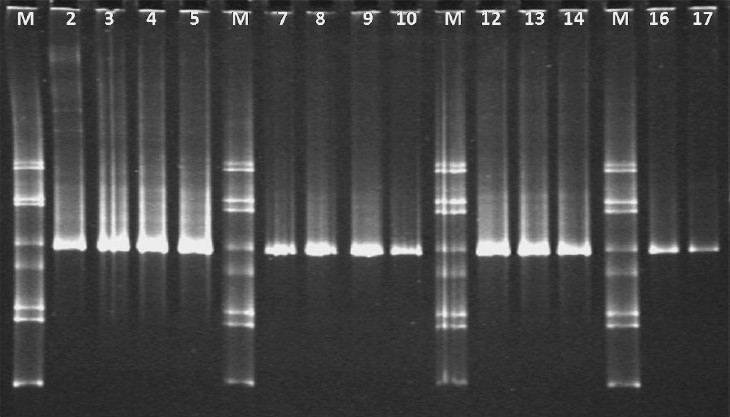
TTGE gel showing bands obtained by electrophoresis of PCR products from neonate faeces using primers targeted at bifidobacterial 16S rDNA sequences. All the samples with single bands are derived from either healthy or rotavirus-infected neonates, while the four lanes showing multiple bands are the bifidobacteria standards used for TTGE. The single bands from faecal samples were sequenced and shown to be *Bifidobacterium longum* subsp. *infantis*. Lanes 1, 6, 11 and 15 are marker. Lanes 2, 4, 7, 9, 12 and 16 are from rotavirus positive stool; remaining lanes from rotavirus negative stool.

In the bacterial TTGE, it is not possible to discriminate between individual species of bifidobacteria. Hence bifidobacteria-specific TTGE was also undertaken. In all the neonates (Control compared to Rota, and Rota+ compared to Rota-) only one band was obtained for bifidobacteria by TTGE (Fig. 2). For identification, bands were excised from the bifidobacterial TTGE gels, cloned and sequenced as described earlier^15^. The newly determined sequences were compared to those available in the public databases, Ribosomal Database Project (RDP) and GenBank®, in order to ascertain their closest relatives. A molecular species was defined as sequences (including reference sequences and clones) with at least 98 per cent similarity. The cloned amplicons were all 99 per cent identical (ranging from 439/442 to 445/447 identity with 0/442 to 1/447 gaps) to *Bifidobacterium longum* subspecies *infantis* (gene for 16S rRNA, partial sequence, DB:ID 186299.2), thus indicating only one species colonizing the gut of these neonates.

**Fig. 3:**
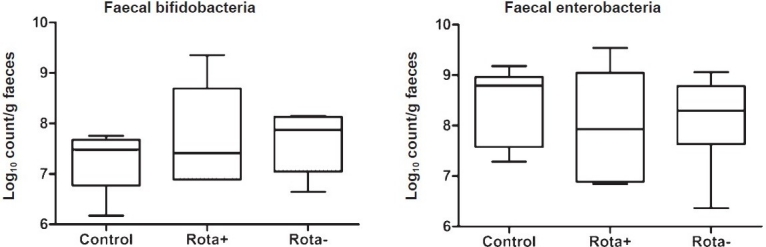
Composite picture showing “box plots” for faecal enterobacteria and bifidobacteria determined by quantitative polymerase chain reaction using primers targeted at 16S rDNA. Bacterial counts are expressed as log_10_ cfu/g faeces. For each set of data, the horizontal line within the box is the median, the box ends are the interquartile range, and the whiskers represent the absolute range. On the X-axis, Control refers to healthy neonates without rotavirus infection at any stage in the first month of life. “Rota+” refers to neonates who had rotavirus infection in the first month of life, while “Rota-” refers to samples from the same neonates after they cleared the rotavirus infection. None of the differences between the groups were statistically significant. Comparisons between Control and Rota+ were made using Mann Whitney U test and comparisons between Rota+ and Rota- were made using Wilcoxon matched pairs test.

As bacterial TTGE showed that bifidobacteria and enterobacteria were the dominant faecal bacteria, we proceeded to quantitate bifidobacteria by real time PCR. We also quantitated enterobacteria as an independent group of bacteria for comparison and internal validation. The median (range) number of bifidobacteria in faeces in the control group was 7.48 (IQR 6.77-7.67) log_10_ cfu/g compared to 7.41 (IQR 6.89-8.69) log_10_ cfu/g in neonates with active rotavirus infection and this was not significantly different. The median (range) number of enterobacteria in faeces in the control group was 8.79 (IQR 7.57-8.96) log_10_ cfu/g was not significantly different from that in the Rota+ group which had a median of 7.92 (IQR 6.88-9.04) log_10_ cfu/g (Fig. 3). Neonates with rotavirus infection did not show significant difference in bifidobacteria counts (log_10_ cfu/g shown as median, range) in paired samples drawn during infected (Rota+, median 7.41, IQR 6.89-8.69) and post-infection (Rota-, median 7.87, IQR 7.04-8.13) periods (Fig. 3). Similarly there were no significant differences in count of enterobacteria (log_10_ cfu/g shown as median, range) between Rota+ (median 7.92, IQR 6.88-9.04) and Rota- (median 8.29, IQR 7.63-8.78) periods.

## Discussion

The present study demonstrates that one species, *Bifidobacterium longum* subspecies *infantis*, predominates in these Indian neonates. It further shows that asymptomatic rotavirus infection at a critical period of development of the intestinal microbiota did not alter bifidobacterial diversity or colonization.

The neonatal gut is sterile *in utero* and becomes colonized with bacteria during and after birth with a succession of bacteria, of which bifidobacteria which colonize the intestine usually during the first and second week of life are thought to confer important health benefits to infants. A number of factors have been documented to affect colonization of the intestine with bifidobacteria, including the time of delivery (premature versus full-term), mode of delivery (vaginal versus Cesarean section), type of feeding (breast-fed versus bottle-fed) and the administration of antibiotics^1^,^4^,^5^,^20^. The factors that determine colonization of the infant intestine with particular microbes continue to be poorly understood, but prior conditioning by environmental or skin bacteria may set the stage for microbes that are established later, by providing appropriate surface molecular expression or luminal growth conditions for these commensal bacteria. Rotavirus infection is common in infants, and in neonates during the first month of life. Studies have repeatedly shown that asymptomatic rotavirus infections may occur^10^–^14^. Since rotavirus is known to damage villus surface epithelial cells in the intestine, it is theoretically possible that in some way it may affect the process of bacterial colonization of the gut that is ongoing in these neonates. Our study, though done in a small number of neonates, shows that such asymptomatic rotavirus infection during the first month of life does not alter the bifidobacterial population in the intestine. We have previously demonstrated, in a community-based molecular study of faecal flora in children, that acute rotavirus gastroenteritis did not significantly alter levels of bifidobacteria in faeces^21^.

Colonization of the gut by bacteria in the neonatal period plays an important role in immune conditioning and may determine host immune responses in later life. The initial gut flora becomes relatively more complex after weaning and eventually a complex flora with around 600 species may be found in the faeces of adults. Lactobacilli and bifidobacteria are established in the intestinal flora of breast-fed infants within the first week of life. Bifidobacteria followed by *Enterobacteriaceae* and *Bacteroides* are quantitatively among the most numerous bacteria in the faeces of infants at the age of one month^20^,^22^. The present study suggests that total bacterial species diversity is low in the first two weeks after birth, although there was high inter-individual variability, closely resembling findings determined using similar techniques in pre-term infants in France^15^.

The nature of bifidobacteria colonizing the infant gut in India has not been described earlier. Using bifidobacteria 16S rDNA PCR followed by TTGE and sequencing the sole bifidobacteria colonizing the neonatal gut in the first month of life in a tertiary care setting in southern India was found to be *Bifidobacterium longum* subspecies *infantis*. Several species of bifidobacteria have been described in the faeces of healthy breast-fed infants, including *B. longum* (various subspecies including *infantis*), *B. breve, B. bifidum* and *B. thermoacidophilum*^4^,^5^,^23^. A study in slightly older infants (aged 1-3 months) than included in the present study suggested that several bifidobacterial species were present in faeces with uncharacterized species accounting for a half, followed by *B. longum* subsp. *infantis* accounting for a quarter, and *B. breve* accounting for one-eighth of faecal bifidobacteria^5^. *B. longum* subsp. *infantis*, is adapted to the optimal use of the nutrients present in breast milk^24^. Their significant role in carbohydrate fermentation and the production of short chain fatty acids may be important to gut development in infancy.

Bifidobacteria, among the intestinal microbiota, play a role in the development of the immune system in the neonate^25^. Interestingly, an earlier study noted that *B. longum* subsp. *infantis* was present in faecal samples of month-old neonates from Ghana but not in similar neonates from New Zealand or the United Kingdom whose faeces showed *B. longum, B. bifidum*, and *B. pseudocatenulatum*^26^. That study, together with our present observations, suggests that there may be differences between developed and developing country infants with regard to intestinal populations of bifidobacteria. It has been suggested that the presence of *B. longum* subsp. *infantis* in developing country neonates offers them protection against immune diseases in later life through immune conditioning in the neonatal period, whereas the other species of bifidobacteria (such as those found in developed country neonates) may not afford such protection^26^. The present findings that *B. longum* subsp. *infantis* is the predominant bifidobacterium in the gut of Indian neonates, and that rotavirus infection within the first month of life does not alter bifidobacterial colonization, are very relevant to human health in India, and will need to be particularly considered in the context of use of probiotic-supplemented foods.

## References

[1] Ramakrishna BS (2007). The normal bacterial flora of the human intestine and its regulation. *J Clin Gastroenterol*.

[2] Guérin-Danan C, Andrieux C, Popot F, Charpilienne A, Vaissade P, Gaudichon C (1997). Pattern of metabolism and composition of the fecal microflora in infants 10 to 18 months old from day care centers. *J Pediatr Gastroenterol Nutr*.

[3] Klaassens ES, Boesten RJ, Haarman M, Knol J, Schuren FH, Vaughan EE (2009). Mixed-species genomic microarray analysis of fecal samples reveals differential transcriptional responses of bifidobacteria in breast- and formula-fed infants. *Appl Environ Microbiol*.

[4] Mariat D, Firmesse O, Levenez F, Guimarăes V, Sokol H, Doré J (2009). The Firmicutes/Bacteroidetes ratio of the human microbiota changes with age. *BMC Microbiol*.

[5] Haarman M, Knol J (2005). Quantitative real-time PCR assays to identify and quantify fecal *Bifidobacterium* species in infants receiving a prebiotic infant formula. *Appl Environ Microbiol*.

[6] Qiao H, Duffy LC, Griffiths E, Dryja D, Leavens A, Rossman J (2002). Immune responses in rhesus rotavirus-challenged BALB/c mice treated with bifidobacteria and prebiotic supplements. *Pediatr Res*.

[7] Phuapradit P, Varavithya W, Vathanophas K, Sangchai R, Podhipak A, Suthutvoravut U (1999). Reduction of rotavirus infection in children receiving bifidobacteria-supplemented formula. *J Med Assoc Thai*.

[8] Mao M, Yu T, Xiong Y, Wang Z, Liu H, Gotteland M (2008). Effect of a lactose-free milk formula supplemented with bifidobacteria and streptococci on the recovery from acute diarrhoea. *Asia Pac J Clin Nutr*.

[9] Saavedra JM, Bauman NA, Oung I, Perman JA, Yolken RH (1994). Feeding of *Bifidobacterium bifidum* and *Streptococcus thermophilus* to infants in hospital for prevention of diarrhoea and shedding of rotavirus. *Lancet*.

[10] Linhares AC, Mascarenhas JD, Gusmão RH, Gabbay YB, Fialho AM, Leite JP (2002). Neonatal rotavirus infection in Belém, northern Brazil: nosocomial transmission of a P[6] G2 strain. *J Med Virol*.

[11] Cunliffe NA, Rogerson S, Dove W, Thindwa BD, Greensill J, Kirkwood CD (2002). Detection and characterization of rotaviruses in hospitalized neonates in Blantyre, Malawi. *J Clin Microbiol*.

[12] Ray P, Sharma S, Agarwal RK, Longmei K, Gentsch JR, Paul VK (2007). First detection of G12 rotaviruses in newborns with neonatal rotavirus infection at All India Institute of Medical Sciences, New Delhi, India. *J Clin Microbiol*.

[13] Ramani S, Sowmyanarayanan TV, Gladstone BP, Bhowmick K, Asirvatham JR, Jana AK (2008). Rotavirus infection in the neonatal nurseries of a tertiary care hospital in India. *Pediatr Infect Dis J*.

[14] Ramani S, Arumugam R, Gopalarathinam N, Mohanty I, Mathew S, Gladstone (2008). Investigation of the environment and of mothers in transmission of rotavirus infections in the neonatal nursery. *J Med Virol*.

[15] Magne F, Abély M, Boyer F, Morville P, Pochart P, Suau A (2006). Low species diversity and high inter-individual variability in faeces of preterm infants as revealed by sequences of 16S rRNA genes and PCR-temporal temperature gradient gel electrophoresis profiles. *FEMS Microbiol Ecol*.

[16] Satokari RM, Vaughan EE, Akkermans AD, Saarela M, de Vos WM (2001). Bifidobacterial diversity in human feces detected by genus-specific PCR and denaturing gradient gel electrophoresis. *Appl Environ Microbiol*.

[17] Pelissier MA, Vasquez N, Balamurugan R, Pereira E, Dossou-Yovo F, Suau A (2010). Metronidazole effects on microbiota and mucus layer thickness in the rat gut. *FEMS Microbiol Ecol*.

[18] Deplancke B, Vidal O, Ganessunker D, Donovan SM, Mackie RI, Gaskins HR (2002). Selective growth of mucolytic bacteria including *Clostridium perfringens* in a neonatal piglet model of total parenteral nutrition. *Am J Clin Nutr*.

[19] Mangin I, Suau A, Magne F, Garrido D, Gotteland M, Neut C (2006). Characterization of human intestinal bifidobacteria using competitive PCR and PCR-TTGE. *FEMS Microbiol Ecol*.

[20] Penders J, Thijs C, Vink C, Stelma FF, Snijders B, Kummeling I (2006). Factors influencing the composition of the intestinal microbiota in early infancy. *Pediatrics*.

[21] Balamurugan R, Janardhan HP, George S, Raghava MV, Muliyil J, Ramakrishna BS (2008). Molecular studies of fecal anaerobic commensal bacteria in acute diarrhea in children. *J Pediatr Gastroenterol Nutr*.

[22] Magne F, Hachelaf W, Suau A, Boudraa G, Bouziane-Nedjadi K, Rigottier-Gois L (2008). Effects on faecal microbiota of dietary and acidic oligosaccharides in children during partial formula feeding. *J Pediatr Gastroenterol Nutr*.

[23] Grönlund MM, Gueimonde M, Laitinen K, Kociubinski G, Grönroos T, Salminen S (2007). Maternal breast-milk and intestinal bifidobacteria guide the compositional development of the *Bifidobacterium* microbiota in infants at risk of allergic disease. *Clin Exp Allergy*.

[24] Sela DA, Chapman J, Adeuya A, Kim JH, Chen F, Whitehead TR (2008). The genome sequence of *Bifidobacterium longum* subsp.*infantis* reveals adaptations for milk utilization within the infant microbiome. *Proc Natl Acad Sci USA*.

[25] Ouwehand A, Isolauri E, Salminen S (2002). The role of the intestinal microflora for the development of the immune system in early childhood. *Eur J Nutr*.

[26] Young SL, Simon MA, Baird MA, Tannock GW, Bibiloni R, Spencely K (2004). Bifidobacterial species differentially affect expression of cell surface markers and cytokines of dendritic cells harvested from cord blood. *Clin Diagn Lab Immunol*.

